# Effects of antenatal corticosteroids on neonatal blood glucose fluctuation in late-preterm infants

**DOI:** 10.3389/fped.2022.1036565

**Published:** 2022-11-11

**Authors:** Cailing Zhou, Wanli Zheng, Meixian Zhang, Tao-Hsin Tung, Linghua Wang, Lizhen Wang

**Affiliations:** Department of Neonatology, Taizhou Enze Medical Center (Group), Taizhou Hospital of Zhejiang Province Affiliated to Wenzhou Medical University, Enze Hospital, Taizhou, China

**Keywords:** late preterm, dexamethasone, gestational age, neonate, hypoglycemia

## Abstract

**Objective:**

To evaluate the effects of antenatal corticosteroids (ACS) on blood glucose fluctuations in late-preterm neonates.

**Methods:**

A retrospective study was performed on 236 neonates with gestational age of 34^+0^ to 36^+6^ weeks who were admitted to the neonatology department of a tertiary general hospital in China's Zhejiang Province between April 2020 and February 2022. The neonates were divided into three groups: complete course, partial course, and control. Primary outcome was the neonatal blood glucose levels within the first 48 h of life.

**Results:**

134 (56.8%) newborns were exposed to a complete course of ACS, 56 (23.7%) had a to a partial course of ACS, and 46 (19.5%) had no exposure to ACS. The patients in the complete course group had the highest proportion of neonatal hypoglycemia (16.4% vs. 3.6% and 6.5%).The patients exposed to a complete course of dexamethasone had significantly lower blood glucose levels within 12 h of birth than the control group, although no significant differences were observed after 24 h. Differences in blood glucose levels were more significant among male infants, although blood glucose curves of the male and female infants remained close to the overall trend.

**Conclusions:**

Blood glucose levels in late-preterm neonates may decrease after ACS administration, especially after exposure to a complete course. The effects are more pronounced in the first 12 h of life, with males being more severely affected; however, the effects on blood glucose levels were not significant 24 h after birth. This can provide a reference for future clinical studies.

## Introduction

Maternal and neonatal health has improved over the past decades; nevertheless, with advances in reproductive assistance, the incidence of preterm birth has not declined (1). The global premature birth rate is estimated at approximately 10% (2). China has the second highest number of premature births worldwide, with >1 million babies born prematurely (1). The rate of premature births in China has reportedly increased by 1.3% annually from 2012 to 2018 (1, 3). Late-preterm infants are defined as those with a gestational age of 34 + 0 to 36 + 6 weeks, and they account for approximately 75% of preterm infants (1). They are at a relatively high risk of common preterm complications, such as respiratory distress syndrome, hypoglycemia, and hyperbilirubinemia (4).

Administration of antenatal corticosteroids (ACS) is an important antenatal intervention to promote fetal lung maturation and improve the prognosis of premature infants. A study by gyamfi Bannerman et al. targeting ACS in late preterm infants (ALPS) showed that antenatal betamethasone treatment significantly improved respiratory outcomes in late preterm infants. However, they reported significantly higher rates of hypoglycemia in these neonates who had experienced ACS (5). Pettit et al. reported the relationship between ACS use and neonatal hypoglycemia, speculating that ACS use may cause maternal hyperglycemia, which in turn leads to fetal pancreatic *β*-cell proliferation/hyperinsulinemia and then neonatal hypoglycemia (6). Pregnant women take large amounts of corticosteroids during pregnancy, and the drugs can pass through the placenta, resulting in inhibitory effects on the adrenal function of the fetus. Neonatal adrenal insufficiency can lead to hypoglycemic symptoms in newborns that also include symptoms such as hypotension and hyponatremia (7). In our study, the mother used dexamethasone prenatally. As far as we know, there is limited research data on changes in blood glucose levels in neonates exposed to dexamethasone. This study focused on neonatal blood glucose fluctuation in these late preterm infants exposed to ACS administration, and we attempted to explore the timing and severity of the effects of ACS on blood glucose in this population.

## Methods

This retrospective study was conducted at a tertiary general hospital in Zhejiang Province, China. Patients were enrolled according to the following criteria: neonatal birth at this hospital, regular glucose level monitoring 48 h after birth, complete maternal ACS dosing data, and accurate maternal and neonatal birth date records. Weeks of gestation for mothers were imputed by last menstrual period. Neonates with congenital inherited metabolic disorders, congenital defects, chromosomal disorders, hospital stays of <2 days, and those who died were also excluded from the study.

**Figure 1:**
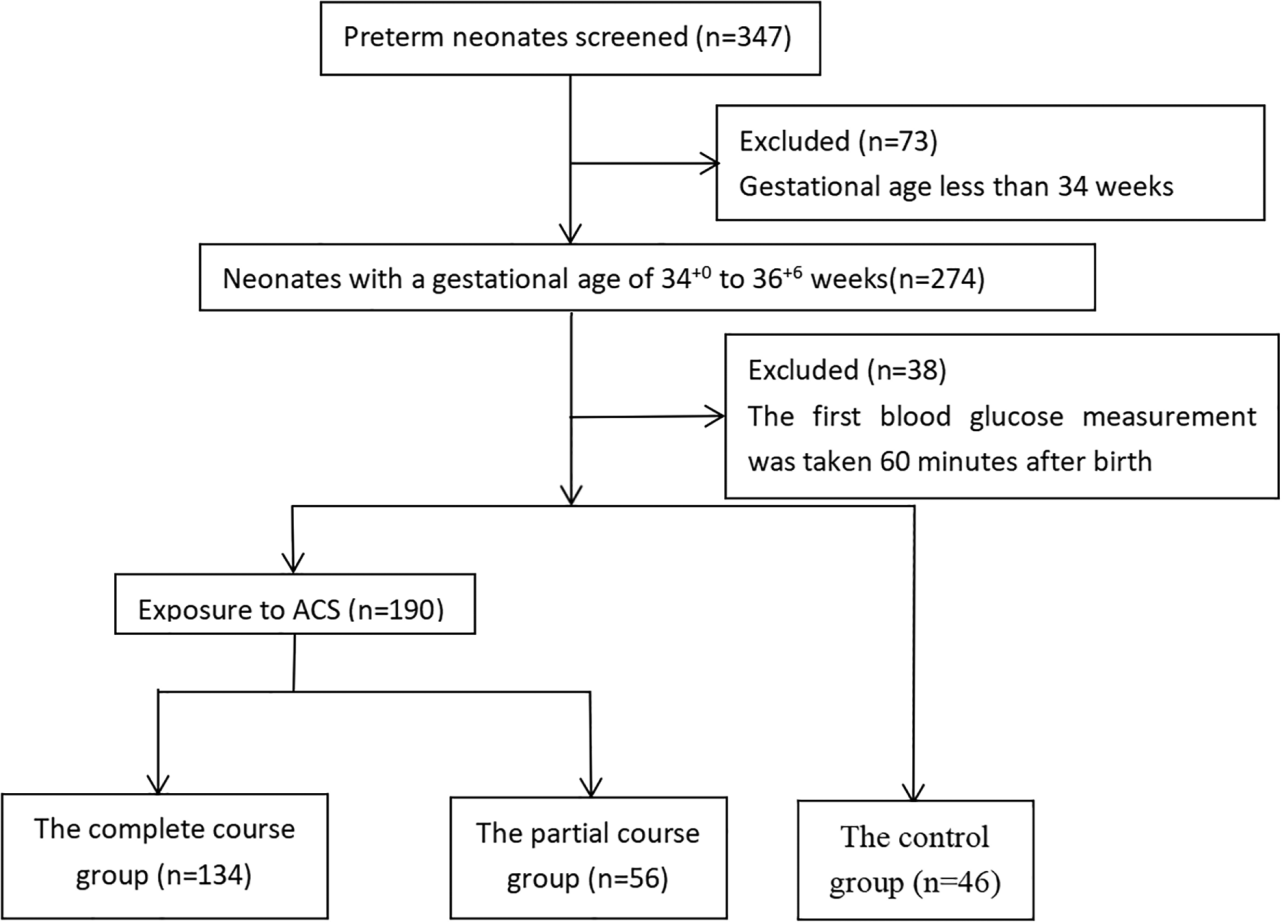
Study participants flowchart.

The research group screened all neonates with a gestational age of 34^+0^ to 36^+6^ weeks who were admitted to the Department of Neonatology between April 2020 and February 2022 (Figure 1). Altogether, 347 premature infants were screened in the study, including 274 premature infants with a gestational age of 34 + 0 to 36 + 6 weeks. 73 preterm neonates with a gestational age <34 weeks were excluded. Because our study aimed to discuss the effects of ACS application on blood glucose level fluctuation within 48 h of birth, the neonates whose first blood glucose level was measured later than 60 min after birth were further excluded because they lack regular blood glucose monitoring.The parents of these premature infants, due to various factors, did not consent to their immediate hospitalization in the neonatal department. There were 38 premature infants excluded due to this criterion. Ultimately, 236 preterm infants were included for statistics. The study protocol was approved by Ethics Committee of Enze Hospital, Taizhou Enze Medical Center (Group)(K20220502).

Demographic data such as the mother's age, mode of delivery, pregnancy history, delivery history, body mass index at delivery, pre-pregnancy history, current pregnancy history, cause of premature birth, neonatal gender, birth weight, head circumference, height, number of fetuses, gestational age at birth, neonatal blood glucose levels and glucose and calorie intake were obtained from the hospital's electronic medical record system.

The application method of ACS in obstetrics of our hospital is as follows: in the study,women with <34 gestational weeks and a risk of preterm birth within 1 week received one course of ACS treatment. Pregnant women with a tendency toward delivery *via* cesarean section and those with premature rupture of membranes or other conditions that might increase fetal risk were also treated with ACS even if the gestational age was >34 weeks (8–12).

The neonates were divided into three groups as follows: the complete course, partial course, and control groups. The complete course of ACS included four 6-mg doses of intramuscular dexamethasone every 12 h, and the partial course included one to three 6-mg doses. The mothers of the neonates in the control group were not exposed to dexamethasone before delivery,because some mothers did not meet the above indications for ACS medication, while some mothers did not have enough time to take medication during emergency delivery. Primary outcome was the neonatal blood glucose levels within the first 48 h of life. Baseline data and blood glucose levels were compared between the three groups.

Neonatal hypoglycemia was defined as a total blood glucose level <2.2 mmol/L (13). Blood glucose levels were measured using a FreeStyle Optium Neo H blood glucose monitor and test paper (Abbott Pharmaceutical Co., Ltd., USA). Each neonate was regularly monitored following admission. Blood glucose levels were screened within 1 h of birth and 2, 4, 12, 24, 36, and 48 h after birth.

### Statistical analyses

A one-way analysis of variance or Mann-Whitney U-test was used to analyze the continuous variable data in the baseline data, and the chi-square test or Fisher's exact test was used to analyze the classified variable data (14). The differences in blood glucose levels between the complete course, partial course, and control groups were analyzed using a one-way analysis of variance. Post-hoc tests were performed between groups. All statistical data were analyzed using the IBM SPSS software (version 25.0; IBM Corp., Armonk, NY, USA). A *P* value < 0.05 on both sides indicated a significant difference.

## Results

Of the remaining 236 eligible preterm infants, 134 (56.8%) were exposed to a complete course of ACS, 56 (23.7%) had a partial course of ACS, and 46 (19.5%) had no exposure to ACS (Table 1).

**Table 1:** Baseline maternal and infant characteristics in the study population.

Baseline demographic	Complete (*n* = 134)	Partial (*n* = 56)	Control (*n* = 46)	*p*-value
Maternal characteristics				
Maternal age (y), mean ± SD	29.6 ± 6.3	28.8 ± 6.6	29.7 ± 5.2	0.648
Nulliparity	51 (38.0)	20 (35.7)	18 (39.1)	0.949
BMI (kg/m^2^) at delivery, mean ± SD	27.8 ± 5.9	27.1 ± 3.8	25.5 ± 5.1	0.054
Chronic hypertension before pregnancy	7 (5.2)	1 (3.8)	0	0.256
Insulin exposure	5 (3.7)	2 (3.6)	0	0.576
Pregestational diabetes	3 (2.2)	0	0	0.580
Obstetric characteristics				
Pregnancy-induced hypertension	21 (15.7)	10 (17.9)	1 (2.2)	0.021
Gestational diabetes	23 (17.2)	10 (17.9)	8 (17.4)	1.000
ICP	5 (2.6)	0	0	0.234
Severe preeclampsia	16 (11.9)	6 (10.7)	0	0.023
PPROM	12 (9.0)	28 (50.0)	22 (47.8)	<0.001
Fetal distress	8 (6.0)	2 (3.6)	1 (2.2)	0.700
Placental abruption	1 (0.7)	1 (1.8)	1 (2.2)	0.399
Placenta praevia	10 (7.5)	3 (5.4)	1 (2.2)	0.485
Vaginal delivery	19 (14.2)	26 (46.4)	19 (41.3)	<0.001
Intrauterine growth restriction	10 (7.5)	1 (1.8)	1 (2.2)	0.248
Gestational age at delivery (wk), mean ± SD	35.0 ± 0.8	34.9 ± 0.8	35.4 ± 0.7	0.001
Infant characteristics				
Male gender	76 (56.7)	29 (51.8)	27 (62.8)	0.778
Twins	63 (47.0)	10 (17.8)	14 (30.4)	<0.001
Birthweight (grams), mean ± SD	2370 ± 430	2433 ± 363	2531 ± 333	0.042
Head circumference, mean ± SD	31.8 ± 1.7	31.9 ± 1.4	32.2 ± 1.3	0.374
Height, mean ± SD	45.9 ± 3.8	46.3 ± 3.3	46.9 ± 3.2	0.083
Ponderal index	2.40 ± 0.28	2.40 ± 0.28	2.40 ± 0.23	0.998
Neonatal hypoglycemia	22 (16.4)	2 (3.6)	3 (6.5)	0.022
Time interval between first ACS administration and delivery (d)	5.5 (3.0,19.8)	0.3 (0.1,0.7)	/	<0.001

BMI, body mass index; ICP, intrahepatic cholestasis of pregnancy; PPROM, preterm premature rupture of membrane.

### Baseline characteristics

A comparison of the baseline characteristics of the three groups of preterm infants is presented in Table 1 Maternal characteristics differed minimally between the groups, with similar maternal age, body mass index at delivery, and medical history prior to pregnancy, followed by similar incidences of gestational diabetes mellitus, fetal distress, placental abruption, placenta previa, and intrauterine growth restriction.Compared with the control group, newborns exposed to ACS had lower birth weight (*p* = 0.042). Women who were treated with dexamethasone were more likely to have pregnancy-induced hypertension (15.7.0% and 17.9% vs. 2.2%, *p* = 0.021), severe preeclampsia (*p* = 0.023), and earlier gestational duration at delivery (35.0 ± 0.8 weeks and 34.9 ± 0.8 weeks vs. 35.4 ± 0.7 weeks). The complete course group was less likely to have preterm premature rupture of membrane (9% vs. 50.0% and 47.8%, *p* < 0.001) and vaginal delivery (14.2% vs. 46.4% and 41.3%, *p* < 0.001). In the complete course group, the incidence of neonatal hypoglycemia was the highest (16.4% vs. 3.6% and 6.5%). Hypoglycemia occurred within 2 h of birth in this study. Table 2 shows that there were no significant differences among the three groups in the intake of sugar and calories during the first 48 h after birth.

**Table 2:** Glucose and calorie intake of the study population within 48 h after birth.

Baseline demographic	Complete (*n* = 134)	Partial (*n* = 56)	Control (*n* = 46)	*p*-value
First 24-hour intake				
Sugar (g/kg), mean ± SD	7.1 ± 0.9	7.0 ± 0.8	7.0 ± 0.7	0.586
Calorie (kcal/kg), mean ± SD	33.9 ± 6.7	34.0 ± 5.4	34.8 ± 4.8	0.695
Second 24-hour intake				
Sugar (g/kg), mean ± SD	7.1 ± 1.4	6.8 ± 1.0	6.8 ± 1.0	0.244
Calorie (kcal/kg), mean ± SD	48.0 ± 8.6	46.6 ± 6.7	46.0 ± 8.2	0.226

### Comparison of blood glucose levels among all groups

**Figure 2:**
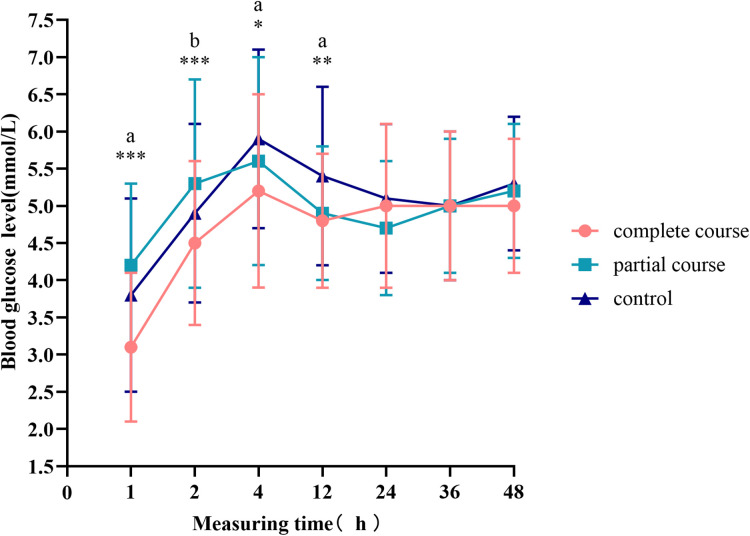
Blood glucose levels at different time points. The curves represent the mean and standard deviation of blood glucose at a specific time point. Comparison of blood glucose levels among all groups, *p < 0.05, **p < 0.01, ***p < 0.001; ^a^*p* < 0.0167 for the comparison of the complete course or partial course group with the control group. ^b^*p* < 0.0167 for the comparison of the complete course group with the partial group.

The mean and standard deviation of the glucose levels are presented in Figure 2. Here, the patients exposed to the complete course of ACS had significantly lower blood glucose levels 12 h after birth than the control group (*p* < 0.001). All the groups had low blood glucose levels at birth, which then continuously increased and peaked at 4 h after birth. After 4 h, the blood glucose levels started to decline. The blood glucose curve of the complete course group rebounded earlier than those of the other two groups at 12 h after birth and then stabilized. The blood glucose levels of the partial course and control groups continued to decrease 12 h after birth; while the blood glucose curve of the control group became stable 24 h after birth, that of the partial course group entered an increasing phase.

The complete course group had the highest proportion of twins in the baseline data. Compared with the control group, the proportion of pregnancy-induced hypertension syndrome in the complete course and partial course groups was higher. Binary logistic regression analysis showed that Pregnancyinduced hypertension, twins and gender had influence on neonatal hypoglycemia (Table 3). However, significant differences were noted as we excluded twins and neonates of mothers with pregnancy-induced hypertension from the general population. The blood glucose fluctuation was similar to that in Figure 2. Within 4 ∼ 24 h, the blood sugar in the control group was the highest. After 24 h, the blood glucose levels of all the groups tended to stabilize.

**Table 3:** The effect of pregnancy-induced hypertension, twins, sex and ACS administration on the risk of hypoglycemia.

	B	S.E,	Wals	OR (95% CI)	*p*-value
Twins	1.173	0.439	7.122	3.231 (1.365-7.647)	0.008
Pregnancy-induced hypertension	0.549	0.549	0.998	1.731 (0.590-5.082)	0.318
Male	1.427	0.461	9.580	4.168 (1.688-10.292)	0.002
ACS administration	0.788	0.781	1.017	2.199 (0.475-10.173)	0.313

CI, confidence interval; OR, odds ratio; SE, standard error; ACS, antenatal corticosteroids.

**Figure 3:**
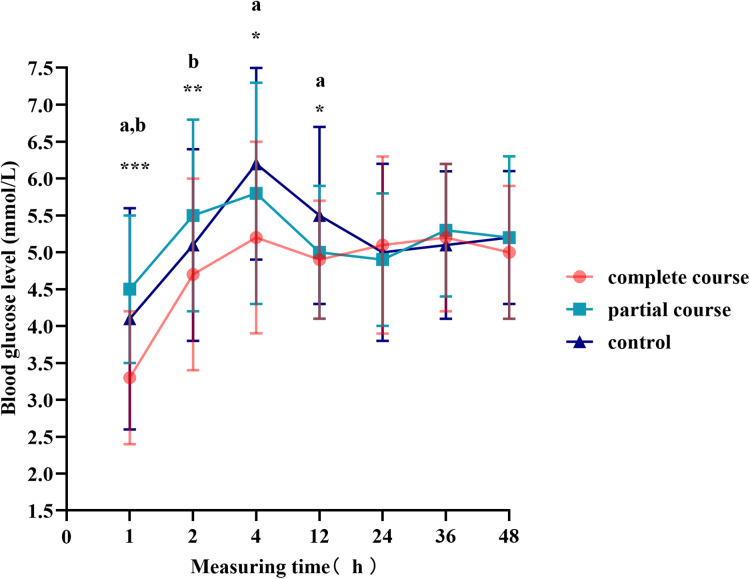
Blood glucose levels of male infants at different time points. Comparison of blood glucose levels of male infants among all groups, *p < 0.05, **p < 0.01, ***p < 0.001; ^a^*p* < 0.0167 for the comparison of the complete or partial course group with the control group; ^b^*p* < 0.0167 for the comparison of the complete course group with partial group.

**Figure 4:**
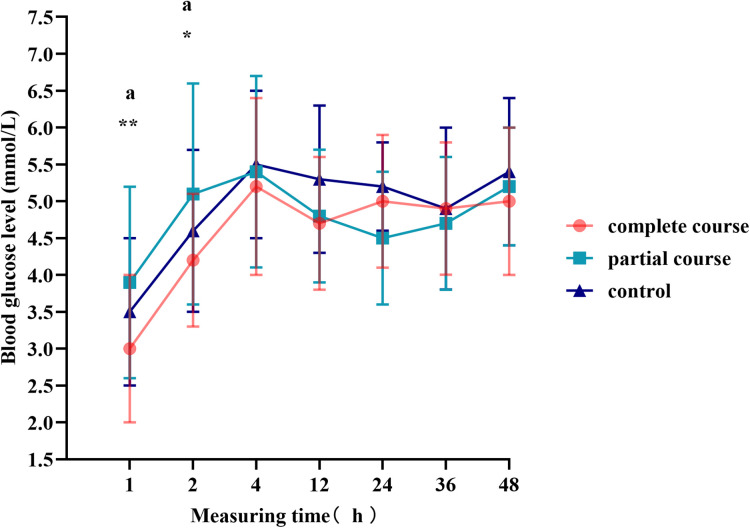
Blood glucose levels of female infants at different time points. Comparison of blood glucose levels of female infants among all groups, *p < 0.05, **p < 0.01, ***p < 0.001; ^a^*p* < 0.0167 for the comparison of the partial course group with the control group.

The *t*-test demonstrated a difference in blood glucose levels between male and female infants; therefore, we divided the total population into male and female groups, performed statistics separately, and plotted the blood glucose fluctuation curves in Figures 3, 4. The blood glucose levels were more significantly different among male infants, with the fluctuation curve consistent with combined male and female population. The difference in the blood glucose levels among the three groups of female infants was reduced, and only the blood glucose levels of the complete course group and partial course group demonstrated statistical significance, within 1 and 2 h of birth, respectively. Meanwhile, the blood glucose levels of the complete course group were higher than those of the partial course group, and those of the three groups were close at 4 h after birth. Since then, the differences in the blood glucose levels among the three groups were not significant; however, the blood glucose curves of the female infants remained close to the overall trend.

## Discussion

### Main findings

The blood glucose levels in late-preterm neonates may decrease, and the probability of neonatal hypoglycemia may increase after ACS administration in mothers, especially after exposure to a complete course of treatment. In the neonates, the effects are more pronounced in the first 12 h of life and vary according to sex. Male infants tend to be more severely affected, although the effects on blood glucose levels are not significant 24 h after birth.

### Clinical significance

At present, several studies on the benefits of ACS application have been reported in the literature. A prospective cohort study of 298 participants in Eastern India in 2021 had revealed that a complete course of ACS reduced the incidence of neonatal hypoglycemia (15). They reported the incidence of hypoglycemia at 12 and 72 h after birth was significantly lower in the complete course group. The mothers of the control group were not given antenatal dexamethasone treatment. This may be because glucose homeostasis is directly affected by dexamethasone, especially in infants born shortly after ACS treatment. We have different results from theirs. However, another retrospective cohort study by Uquillas et al. revealed a greater risk of hypoglycemia in newborns delivered by mothers receiving ACS (16). Mothers of newborns hospitalized with hypoglycemia in the NICU had a higher incidence of ACS (odds ratio adj 4.71, *p* adj = 0.01). Our study results are consistent with those of Uquillas et al. Here, the blood glucose levels of neonates with exposure to a complete course of ACS remained the lowest within 12 h, while those of neonates without ACS exposure remained peaked during the period of 4–12 h.

### Interpretation

In our study, the incidence of hypoglycemia was higher in neonates exposed to ACS than in neonates without exposure to ACS and was relatively lower compared with those in some other antenatal betamethasone studies (4, 17). The pathophysiological mechanism of ACS-related neonatal hypoglycemia has not yet been fully elucidated. In the first 2 h, the blood glucose levels of the partial course groups were the highest, and the group with the highest glucose levels after 2 h was the control group. According to some experiments, muscle injection of dexamethasone reached peak plasma concentration at 6 h, and the median time to return to baseline blood glucose levels after dexamethasone treatment was 33.1 h (18). Hyperglycemia begins at approximately 3 h with the long-acting glucocorticoid dexamethasone, peaks at approximately 9–10 h, and resolves 48 h after discontinuation (19). We speculated that the fluctuation in blood glucose levels was related to the time required for maternal blood glucose and insulin levels to peak after antenatal dexamethasone application, time to delivery after dexamethasone application, and effect of maternal hyperglycemia on neonatal insulin levels (4, 13, 18, 20). After umbilical cord ligation, the glucose and dexamethasone transmitted by the umbilical cord were immediately terminated, and the neonates left the dexamethasone and maternal hyperglycemic environment and entered self-regulation. The stress state of delivery and postnatal energy through oral and intravenous administration may cause an increase in postnatal blood glucose.

### Research implications

It was generally believed that long-term and severe hypoglycemia might affect the neonatal nervous system. Neonatal hypoglycemia may lead to seizures and may progress to coma, death, or severe neurodevelopmental abnormalities. These findings can be observed in patients with severe and persistent hypoglycemia (21). A systematic review has revealed that neonatal hypoglycemia is associated with a 2- to 3-fold increased risk of specific cognitive deficits in early childhood, including executive dysfunction and visual–motor dysfunction (22). A randomized clinical trial by Taygen Edwards et al. reported that compared with patients without hypoglycemia at birth, those children who experienced hypoglycemia in the early life had a significantly higher incidence rate of neurosensory impairment at the age of 2 years (23% vs. 18%) (23). In recent years, studies have found that transient asymptomatic hypoglycemia can also cause neurological sequelae. McKinlay et al. showed that children with a history of hypoglycemia within 12 h of birth were at a greater risk for combined neurosensory impairment (24). Kaiser Jr et al. examined the association of hypoglycemia within 3 h of birth with poor academic performance, proposing that early transient hypoglycemia may correlate with poor academic performance at 10 years (25). In our study, although hypoglycemia caused by antenatal dexamethasone treatment was mostly transient, it might carry the potential risks mentioned above. Therefore, early detection and management of neonatal hypoglycemia are important. In addition, transient neonatal hypoglycemia has been associated with post-natal hyperinsulinemia (26). By studying the fluctuation of blood glucose, we can indirectly reveal changes in insulin to some extent. The blood glucose levels of all three groups entered the steady state at 24 h, and no statistically significant difference was observed, indicating that blood glucose regulation was completed within 24 h as well as that of insulin. In subsequent studies, neonatal insulin levels may be analyzed to verify the effects of prenatal corticosteroids on neonatal insulin levels.

### Strengths and limitations

To the best of our knowledge, this is the first study to examine the effects of ACS on blood glucose fluctuation in preterm infants in China as some studies only focused on abnormal outcomes and did not consider the effects of the blood glucose fluctuation curve. In this study, the blood glucose levels of new infants were regularly monitored, and the curve of blood glucose fluctuation was observed, including the differences between male and female infants. This can provide a reference for future clinical studies.

Nevertheless, this study had some limitations. First, it was limited to 48-h blood glucose monitoring without long-term monitoring and was not representative of long-term follow-up results. Second, only changes in blood glucose levels were observed, and no changes in blood glucose-regulating hormones, such as insulin, were observed. Third, the measurement specimen used was peripheral blood from the capillaries as collecting venous blood is difficult. Considering ethical issues, the blood glucose levels were measured with peripheral blood, and many studies have reported that the difference between peripheral blood glucose and venous blood glucose is insignificant and does not affect the blood glucose curve (27–30).

## Conclusion

Antenatal dexamethasone treatment for mothers may have a temporary negative effect on neonatal blood glucose levels in late-preterm infants within 24 h of birth. Therefore, regular glucose monitoring is recommended for late-preterm infants with a history of ACS exposure, especially for the first 24 h after birth.

## Data Availability

The original contributions presented in the study are included in the article/Supplementary Material, further inquiries can be directed to the corresponding author/s.
